# Resistance to grain protectants and synergism in Pakistani strains of *Sitophilus oryzae* (Coleoptera: Curculionidae)

**DOI:** 10.1038/s41598-022-16412-y

**Published:** 2022-07-20

**Authors:** Tiyyabah Khan, Muhammad Saleem Haider, Hafiz Azhar Ali Khan

**Affiliations:** 1 Department of Plant Pathology, University of the Punjab, Lahore, Pakistan; 2 Department of Entomology, University of the Punjab, Lahore, Pakistan

**Keywords:** Entomology, Environmental impact

## Abstract

The widespread use of insecticides for the management of insect pests in storage facilities and food industries have caused insecticide resistance a frequent issue worldwide. Nonetheless, this issue has been little explored in Pakistan that resulted in control failures and increased dosage of insecticides. In the present study, insecticide resistance to chlorpyrifos-methyl, pirimiphos-methyl, permethrin and spinosad was surveyed in five field strains of *Sitophilus oryzae*: FSD-SO, GJR-SO, DGK-SO, MTN-SO and BWP-SO, collected from five different localities of Punjab, Pakistan, and contrasted with an insecticide susceptible reference strain (Lab-SO). Dose-mortality bioassays were performed in glass vials containing insecticide-treated rice grains, and lethal doses (LD_50_ and LD_95_) were calculated and compared using the ratio tests. In comparison to the Lab-SO strain at LD_50_ and LD_95_ levels, field strains exhibited: 24.51 to 52.80 and 36.55 to 69.31 resistance ratios (RRs), respectively, for chlorpyrifos-methyl; 15.89 to 45.97 and 55.12 to 194.93 RRs, respectively, for pirimiphos-methyl; 39.76 to 108.61 and 61.33 to 130.12 RRs, respectively, for permethrin; 4.23 to 27.50 and 6.28 to 41.00 RRs, respectively, for spinosad. In the synergism experiments using the Lab-SO and the most resistant strains against each insecticide, the enzyme inhibitors (PBO and DEF) failed to synergize toxicity of insecticides in the Lab-SO strain; however, toxicity of chlorpyrifos-methyl, pirimiphos-methyl and permethrin significantly enhanced in the resistant strains of *S. oryzae*, suggesting possibility of metabolic mechanism of resistance. In addition, activities of detoxification enzymes (CarE, MFO and GST) were significantly higher in resistant strains compared to the Lab-SO strain. The results revealed presence of insecticide resistance in field strains of *S. oryzae* that necessitate the need to develop a resistance management strategy.

## Introduction

Tropical and subtropical climatic conditions usually provide an ideal environment for the growth and population expansion of stored insect pests in storage facilities. Besides direct damage to stored commodities during feeding, stored insect pests are also linked with dissemination of fungal spores during their continuous movement inside and/or over the stored commodities^1,2^. Since the 1960s, insecticides have been used extensively for the management of insect pests of stored commodities in various storage facilities such as granaries, warehouses and flour mills. Insecticides are mainly applied as aerosols, fumigants, grain or residual treatment in order to ensure long-term protection to stored insect pests^3^. The use of insecticides is amongst the major tools to manage stored insects, particularly in Pakistan, which is also linked with environmental and public health concerns^4,5^.

The rice weevil, *Sitophilus oryzae* (Linnaeus), is one of major insect pests of stored commodities causing economic damages to a variety of products such as cereal grains, flour and dry fruits^1^. Insecticidal control of *S. oryzae* has been one of the major tools, which include insecticides from carbamate (e.g., carbaryl), pyrethroid (e.g., permethrin, deltamethrin) and organophophate (e.g., chlorpyrifos-methyl, pirimiphos-methyl) classes^6–9^.

The widespread use of DDT (dichlorodiphenyltrichloroethane) up to the 1980’s and synthetic organophosphates and pyrethroids afterward in an effort to control insect pests in stored commodities have resulted in the evolution of insecticide resistance and cross-resistance that ultimately caused economic losses in storage facilities besides environmental concerns^8^. Chlorpyrifos-methyl, pirimiphos-methyl and permethrin have been in use in Pakistan for the management of stored insect pests including *S. oryzae* for more than the last two decades^4,10^. Spinosad is not yet registered but have potential as grain protectant in Pakistan^11,12^. The use of insecticides against stored insect pests has made development of insecticide resistance a frequent issue in the successful management of these pests worldwide^7,13–15^. Recently, resistance to pirimiphos-methyl and permethrin^4^, and deltamethrin^16^ have been reported in different field strains of *Trogoderma granarium* (Everts) from Pakistan. Therefore, there is a probability of insecticide resistance in other stored insects such as *S. oryzae* that usually inhabit the same environment. However, no attempt has so far been made to check the status of insecticide resistance in Pakistani strains of *S. oryzae* despite the long term usage of insecticides.

Activation of detoxifying enzymes is assumed as one of the major factors responsible for insecticides resistance, which can be initially assessed using enzyme inhibitors in insecticidal bioassays^17–19^. For instance, the enzyme inhibitors S,S,S-tributyl phosphorotrithioate (an esterase specific inhibitor) and piperonyl butoxide (an inhibitor of cytochrome P450 monooxygenases and of esterases) have long been used to preliminary assessment of the involvement of metabolic enzymes in the development of insecticide resistance in different insect pests^15,20–22^.

Evolution of insecticide resistance is inevitable when the use of insecticide against stored insects is a major control measure. Presence of insecticide resistance in Pakistani strain of *S. oryzae* is still unclear, although suspected following the recent control failures. The present study reports insecticide resistance, synergism and metabolic-mechanism of resistance in Pakistani strains of *S. oryzae.*

## Materials and methods

### Insects

Between June and July of 2020, five field strains of *S. oryzae* were collected from rice-storage facilities in five different cities across Punjab province: Faisalabad (31.4504° N, 73.1350^o^ E), Gujranwala (32.1877° N, 74.1945° E), Dera Ghazi Khan (30.0489° N, 70.6455° E), Multan (30.1575° N, 71.5249° E), and Bahawalpur (29.3544° N, 71.6911° E). These strains were coded as FSD-SO, GJR-SO, DGK-SO, MTN-SO, and BWP-SO, respectively. At least 300 adults were used to develop each of the field strain in the laboratory of Entomology. A reference susceptible strain (Lab-SO) maintained at the Department of Entomology, University of the Punjab, Lahore, for over nine years without exposure to any chemical/pesticide was used in resistance screening to insecticides in field strains. The Lab-SO strain has showed susceptibility to different insecticides in the present and previous studies^11,23^. All strains were grown in clean glass jars (2-L capacity) containing pesticide/infestation free rice grains under controlled conditions of 27 °C, 65% relative humidity and without lighting.

### Chemicals

Four technical-grade insecticides: spinosad (a bacterial-based insecticide; 94.2%), chlorpyrifos-methyl, pirimiphos-methyl (organophosphates; 99%) and permethrin (a pyrethroid; 99.5%) were used in resistance screening and synergism bioassays (ChemService Inc. West Chester, PA, USA). Two synergists: piperonyl butoxide (PBO) and S,S,S-tributyl phosphorotrithioate (DEF) (ChemService Inc. West Chester, PA, USA) were used in synergism bioassays.

### Bioassays

The method of insecticidal bioassays adapted from recently reported insecticide resistance studies by Khan^23^ and Khan^4^. Six different concentrations, causing > 0 and < 100% mortality, of each insecticide and a control (acetone alone) were used for bioassays with each strain. The range of concentration of insecticides against the Lab-SO strain was comprised of: 0.125–4 mg a.i./kg of grains of chlorpyrifos-methyl, pirimiphos-methyl or permethrin, and 0.05–1.6 mg a.i./kg of grains of spinosad. In the case of field strains of *S. oryzae*, the range of concentrations used were: 2–64 mg a.i./kg of grains of chlorpyrifos-methyl and pirimiphos-methyl, 3–96 mg a.i./kg of grains of permethrin, and 0.4–12.8 mg a.i./kg of grains of spinosad. Clean rice (1 kg), purchased from the local market, were mixed with a solution of a particular insecticide concentration (1 mL) using an AG4 air brush. For the purpose to ensure even distribution of insecticide solution onto the whole grains, the treated grains were shifted into clean glass jars and manually shaken for ten minutes. The grain treatment with each concentration of insecticides was replicated five times by making fresh insecticide solutions each time. The same procedure was followed to prepare control treatment by using only acetone. Bioassay glass-vials (20-mL) were prepared by taking ten grams of rice grains from each concentration-treated or control lot of rice grains and introducing ten freshly emerged adults of *S. oryzae* into the vials per concentration, in five replicates. The top of each vial was sealed with muslin cloth to bar insects escaping. The vials were left under the controlled environment with 27 °C, 65% relative humidity and darkness. The vials were checked after seven days of insects’ exposure into the vials in order to confirm mortality if they showed no movement on disturbance with a camel-hair-brush.

In synergism bioassays, the same bioassay procedure was followed except the insects were exposed to synergist-coated glass vials before introducing into the glass vials having treated rice grains. The vials for synergism bioassays were coated with 1 mL solution of PBO or DEF (1 mg/ml of acetone) and the insects were introduced into the dried synergist residue vials for 1 h before their use in insecticide bioassays^4,15^. Exposure of Lab-SO and field strains of *S. oryzae* to the said concentration of either synergist alone in our preliminary bioassays resulted in no mortality. All the experiments comply with local and national guidelines.

Biochemical analyses for carboxylesterase (CarE), mixed function oxidase (MFO), and glutathione-S-transferase in different strains of *S. oryzae* were performed following the methodology of Khan et al.^24,25^:

Six replicates of adult weevils were used for enzyme analyses. Sodium phosphate buffer (01 mL; pH7.8; 0.1M) was used to prepare homogenate of these insects, followed by centrifugation for 10 min at 10,000 × g. After the centrifugation, large fragments of insects’ body were removed. The supernatant was then used for determining the activities of MFO, CarE, and GST by using the protocols described by Yang et al.^26^, Gao et al.^27^, and Bradford^28^ protocol for total proteins analysis. Analyses were performed in 96-well microtiter plates in six replicates.

### Data analyses

Data from insecticidal bioassays and synergism experiments were analyzed as outlined in our previous report (p. 2–3)^4^:

“Mean mortality counts from dose-mortality bioassays of each strain against each rate of chlorpyrifos-methyl, pirimiphos-methyl, permethrin or spinosad were corrected, if needed, for mortality counts in the control treatment^29^. Mortality data were analyzed by Probit analysis using the software PoloPlus^30^ to determine lethal doses (LD_50_ and LD_95_) and 95% confidence intervals (CIs). Any two LD_50_ or LD_95_ values were considered significantly different if their 95% CI values did not overlap^31^. Ratio tests were performed to compare LD_50_ and LD_95_ values of field strains with those of the corresponding laboratory reference strain, and considered significantly different if 95% CI of the ratio did not include one^32^. The same criterion was applied to determine the significance of LD values of chlorpyrifos-methyl, pirimiphos-methyl, permethrin and spinosad with or without synergist in synergism experiments”^23,33^.

## Results

Toxicity responses of the Lab-SO and field strains of *S. oryzae* in dose-mortality bioassays to insecticide tested are presented in the Table 1. LD_50_ and LD_95_ values of all the tested insecticides were lower in the Lab-SO strain when compared with those of the field strains. LD_50_ and LD_95_ values estimated in the Lab-SO strain were: 0.51 and 2.36 mg/kg of grains for chlorpyrifos-methyl, 0.65 and 2.28 mg/kg of grains for pirimiphos-methyl, 0.38 and 2.33 mg/kg of grains for permethrin, and 0.22 and 0.85 mg/kg of grains for spinosad, respectively. These values were served as reference points for estimating variation in toxicity and resistance detection to insecticides in field strains.

**Table 1:** Toxicity of insecticides in a laboratory and field strains of *Sitophilus oryzae.*

Insecticide	Strain	LD_50_* (95% CI) (mg/kg of grain)	LD_95_** (95% CI) (mg/kg of grain)	Fit of probit line				LD_50_ ratio (95% CI)^£^	LD_95_ ratio (95% CI)^£^
				Intercept (± SE)	Slope (± SE)	*χ*^2^ (df = 4)	*p*		
Chlorpyrifos-methyl	Lab-SO	0.51 (0.43–0.61)	2.36 (1.79–3.44)	0.71 (0.11)	2.49 (0.23)	2.91	0.57	–	–
	FSD-SO	13.72 (11.08–17.10)	115.69 (75.98–212.86)	− 2.02 (0.22)	1.78 (0.18)	0.91	0.92	26.90 (20.21–35.20)	49.02 (26.93–89.61)
	GJR-SO	14.92 (12.34–18.15)	91.26 (64.29–149.60)	− 2.45 (0.25)	2.09 (0.20)	2.09	0.72	29.25 (22.37–37.60)	38.67 (22.85–65.71)
	DGK-SO	12.50 (9.95–15.77)	126.17 (79.91–246.05)	− 1.80 (0.21)	1.64 (0.17)	3.24	0.52	24.51 (18.22–32.40)	53.47 (28.22–101.74)
	MTN-SO	26.93 (22.05–33.94)	163.58 (108.14–301.47)	− 3.00 (0.30)	2.10 (0.22)	2.78	0.60	52.80 (39.74–68.97)	69.31 (38.21–126.27)
	BWP-SO	16.31 (10.33–20.30)	86.26 (49.33–245.15)	− 2.49 (0.25)	2.14 (0.20)	6.56	0.16	31.98 (22.00–36.86)	36.55 (21.81–61.51)
Pirimiphos-methyl	Lab-SO	0.65 (0.47–0.89)	2.28 (1.50–4.94)	0.56 (0.11)	3.02 (0.28)	7.67	0.10	–	–
	FSD-SO	21.28 (17.75–25.94)	110.85 (78.34–181.92)	− 3.05 (0.30)	2.30 (0.23)	1.73	0.79	32.74 (25.58–41.74)	48.62 (29.57–80.16)
	GJR-SO	10.33 (8.05–13.16)	125.67 (77.13–261.88)	− 1.54 (0.19)	1.51 (0.17)	2.16	0.71	15.89 (11.88–21.18)	55.12 (28.62–106.49)
	DGK-SO	23.14 (19.12–28.59)	133.73 (91.55–231.69)	− 2.95 (0.30)	2.16 (0.22)	2.02	0.73	35.60 (27.58–45.76)	58.65 (34.46–100.13)
	MTN-SO	29.88 (22.56–43.05)	444.45 (219.49–1394.11)	− 2.07 (0.23)	1.40 (0.17)	1.62	0.81	45.97 (32.26–65.26)	194.93 (77.03–494.70)
	BWP-SO	19.28 (15.00–25.74)	259.09 (143.20–649.38)	− 1.87 (0.21)	1.46 (0.17)	3.17	0.53	29.66 (21.74–40.32)	113.64 (51.98–249.13)
Permethrin	Lab-SO	0.38 (0.31–0.46)	2.33 (1.70–3.63)	0.88 (0.11)	2.10 (0.21)	1.95	0.74	–	–
	FSD-SO	38.68 (31.08–50.09)	303.17 (188.10–619.02)	− 2.92 (0.30)	1.84 (0.20)	3.17	0.53	101.79 (74.25–138.56)	130.12 (65.23–260.38)
	GJR-SO	41.27 (29.32–64.44)	246.64 (129.12–403.10)	− 3.42 (0.35)	2.12 (0.23)	6.04	0.20	108.61 (80.81–161.20)	105.85 (74.64–195.51)
	DGK-SO	21.27 (19.59–26.68)	285.53 (164.59–664.15)	− 1.94 (0.23)	1.46 (0.17)	2.17	0.70	55.97 (40.29–77.21)	122.55 (56.57–266.33)
	MTN-SO	37.33 (27.43–49.63)	378.02 (218.60–876.38)	− 2.57 (0.27)	1.64 (0.19)	0.31	0.99	98.24 (70.45–135.96)	162.24 (75.12–351.86)
	BWP-SO	15.11 (12.04–18.88)	142.89 (93.17–266.98)	− 1.99 (0.23)	1.69 (0.18)	1.66	0.80	39.76 (29.27–53.62)	61.33 (32.46–116.24)
Spinosad	Lab-SO	0.22 (0.17–0.28)	0.85 (0.60–1.49)	1.84 (0.19)	2.80 (0.26)	4.36	0.36	–	–
	FSD-SO	6.05 (4.43–9.17)	34.85 (18.97–112.75)	− 1.69 (0.17)	2.16 (0.24)	5.05	0.28	27.50 (21.01–36.11)	41.00 (22.72–73.63)
	GJR-SO	1.64 (1.27–2.10)	6.12 (4.25–11.14)	− 0.61 (0.11)	2.87 (0.27)	4.79	0.31	7.45 (5.93–9.34)	7.20 (4.73–10.88)
	DGK-SO	1.51 (1.22–1.85)	10.82 (7.59–17.91)	− 0.34 (0.09)	1.92 (0.20)	2.16	0.71	6.86 (5.28–8.94)	12.73 (7.59–21.23)
	MTN-SO	1.84 (1.49–2.26)	13.79 (9.56–23.24)	− 0.50 (0.10)	1.88 (0.19)	1.06	0.90	8.36 (7.35–10.21)	16.22 (12.56–20.39)
	BWP-SO	0.93 (0.75–1.13)	5.34 (3.92–8.37)	0.06 (0.03)	2.17 (0.24)	1.44	0.84	4.23 (3.26–5.52)	6.28 (3.91–10.06)

*Lethal dose to kill 50% insects.

**Lethal dose to kill 95% insects.

^£^Significant ratios based on the ratio test, i.e., 95% CI of the ratio did not include 1^32^. Ratio tests for analyzing presence of resistance to different insecticides in field strains of *Sitophilus oryzae* in comparison to the Lab-SO strain at LD_50_ and LD_95_ levels.

Field strains exhibited 12.50 to 26.93 mg/kg of grains LD_50s_ for chlorpyrifos-methyl; 10.33 to 29.88 mg/kg of grains LD_50s_ for pirimiphos-methyl; 15.11 to 41.27 mg/kg of grains LD_50s_ for permethrin; 0.93 to 6.05 mg/kg of grains LD_50s_ for spinosad (Table 1). The LD_95_ values ranged from 86.26 to 163.58 mg/kg of grains for chlorpyrifos-methyl; 110.85 to 444.45 mg/kg of grains for pirimiphos-methyl; 142.89 to 378.02 mg/kg of grains for permethrin; 5.34 to 34.85 mg/kg of grains for spinosad. Based on LD_50_ values, the MTN-SO strain was the least susceptible to chlorpyrifos-methyl, while three of the field strains (MTN-SO, DGK-SO and FSD-SO) were less susceptible to pirimiphos-methyl compared to rest of the strains. In the case of permethrin, GJR-SO, FSD-SO and MTN-SO were less susceptible compared with BWP-SO and DGK-SO strains. The FSD-SO strain was the least susceptible field strain to spinosad (Table 1).

Ratio tests revealed significant differences in between the Lab-SO strain and any of the field strains compared both at LD_50_ and LD_95_ levels (Table 2). In comparison to the Lab-SO strain at LD_50_ and LD_95_ levels, field strains exhibited: 24.51 to 52.80 and 36.55 to 69.31 resistance ratios, respectively, for chlorpyrifos-methyl; 15.89 to 45.97 and 55.12 to 194.93 resistance ratios, respectively, for pirimiphos-methyl; 39.76 to 108.61 and 61.33 to 130.12 resistance ratios, respectively, for permethrin; 4.23 to 27.50 and 6.28 to 41.00 resistance ratios, respectively, for spinosad (Table 1).

**Table 2:** Synergism of insecticides toxicity in laboratory and field strains of *Sitophilus oryzae.*

Insecticide	Strain	Insects tested	LD_50_ (95% CI) (mg/kg of grain)	LD_95_ (95% CI) (mg/kg of grain)	Fit of probit line				Synergism ratio^a^ at LD_50_ (95% CI)	Synergism ratio^a^ at LD_95_ (95% CI)
					Intercept	Slope (SE)	χ^2^ (df = 4)	*P*		
Chlorpyrifos-methyl	Lab-SO	350	0.51 (0.43–0.61)	2.36 (1.79–3.44)	0.71 (0.11)	2.49 (0.23)	2.91	0.57		
Chlorpyrifos-methyl + PBO	Lab-SO	350	0.63 (0.52–0.77)	4.13 (2.92–6.74)	0.40 (0.09)	2.01 (0.19)	2.79	0.59	0.81 (0.63–1.06)	0.57 (0.34–1.09)
Chlorpyrifos-methyl + DEF	Lab-SO	350	0.50 (0.37–0.69)	2.59 (1.62–5.91)	0.69 (0.11)	2.32 (0.22)	5.74	0.22	1.02 (0.79–1.31)	0.91 (0.56–1.47)
Chlorpyrifos-methyl	MTN-SO	350	26.93 (22.05–33.94)	163.58 (108.14–301.47)	− 3.00 (0.30)	2.10 (0.22)	2.78	0.60		
Chlorpyrifos-methyl + PBO	MTN-SO	350	4.59 (3.76–5.48)	21.07 (15.97–31.41)	− 1.64 (0.23)	2.49 (0.27)	0.91	0.92	5.87 (4.41–7.79)*	7.76 (4.25–14.17)*
Chlorpyrifos-methyl + DEF	MTN-SO	350	6.38 (5.28–7.63)	32.28 (24.09–48.71)	− 1.88 (0.22)	2.34 (0.24)	1.21	0.87	4.22 (3.18–5.60)*	5.07 (2.75–9.33)*
Pirimiphos-methyl	Lab-SO	350	0.65 (0.47–0.89)	2.28 (1.50–4.94)	0.56 (0.11)	3.02 (0.28)	7.67	0.10		
Pirimiphos-methyl + PBO	Lab-SO	350	0.53 (0.38–0.73)	2.29 (1.31–8.18)	0.71 (0.12)	2.59 (0.24)	5.71	0.22	1.23 (0.97–1.54)	0.99 (0.65–1.51)
Pirimiphos-methyl + DEF	Lab-SO	350	0.72 (0.60–0.85)	3.32 (2.49–4.90)	0.36 (0.09)	2.47 (0.23)	0.81	0.93	0.90 (0.72–1.15)	0.69 (0.45–1.06)
Pirimiphos-methyl	MTN-SO	350	29.88 (22.56–43.05)	444.45 (219.49–1394.11)	− 2.07 (0.23)	1.40 (0.17)	1.62	0.81		
Pirimiphos-methyl + PBO	MTN-SO	350	3.47 (1.99–4.99)	26.14 (15.59–74.54)	− 1.05 (0.20)	1.88 (0.23)	5.22	0.27	8.61 (5.70–12.97)*	17.00 (6.35–45.53)*
Pirimiphos-methyl + DEF	MTN-SO	350	4.15 (3.35–4.98)	19.89 (14.96–30.07)	− 1.49 (0.22)	2.42 (0.27)	2.57	0.63	7.20 (4.96–10.46)*	22.35 (8.63–57.86)*
Permethrin	Lab-SO	350	0.38 (0.31–0.46)	2.33 (1.70–3.63)	0.88 (0.11)	2.10 (0.21)	1.95	0.74		
Permethrin + PBO	Lab-SO	350	0.53 (0.43–0.65)	3.73 (2.63–6.10)	0.54 (0.10)	1.94 (0.19)	2.25	0.69	0.72 (0.54–1.03)	0.62 (0.36–1.09)
Permethrin + DEF	Lab-SO	350	0.48 (0.39–0.57)	2.76 (2.02–4.31)	0.69 (0.11)	2.15 (0.21)	2.48	0.65		
Permethrin	GJR-SO	350	41.27 (29.32–64.44)	246.64 (129.12–403.10)	− 3.42 (0.35)	2.12 (0.23)	6.04	0.20		
Permethrin + PBO	GJR-SO	350	26.35 (21.43–32.97)	201.10 (132.09–369.66)	− 2.65 (0.27)	1.86 (0.19)	2.99	0.56	1.57 (1.16–2.12)*	1.23 (1.03–2.49)*
Permethrin + DEF	GJR-SO	350	23.01 (19.33–27.58)	112.55 (82.75–172.37)	− 3.25 (0.31)	2.39 (0.23)	2.49	0.65	1.57 (1.36–2.37)*	2.19 (1.18–4.06)*
Spinosad	Lab-SO	350	0.22 (0.17–0.28)	0.85 (0.60–1.49)	1.84 (0.19)	2.80 (0.26)	4.36	0.36		
Spinosad + PBO	Lab-SO	350	0.28 (0.24–0.34)	1.35 (1.02–1.86)	1.32 (0.15)	2.45 (0.23)	1.66	0.80	0.79 (0.60–0.96)	0.63 (0.40–0.98)
Spinosad + DEF	Lab-SO	350	0.23 (0.18–0.31)	1.06 (0.71–2.05)	1.59 (0.17)	2.51 (0.23)	4.77	0.31	0.96 (0.75–1.20)	0.80 (0.52–1.25)
Spinosad	FSD-SO	350	6.05 (4.43–9.17)	34.85 (18.97–112.75)	− 1.69 (0.17)	2.16 (0.24)	5.05	0.28		
Spinosad + PBO	FSD-SO	350	6.36 (5.07–8.42)	47.74 (28.86–103.35)	− 1.51 (0.15)	1.88 (0.21)	3.04	0.55	0.95 (0.68–1.33)	0.73 (0.33–1.63)
Spinosad + DEF	FSD-SO	350	5.75 (4.57–7.63)	48.48 (28.86–106.83)	− 1.35 (0.14)	1.78 (0.20)	3.82	0.43	1.05 (0.75–1.47)	0.72 (0.32–1.63)

^a^Synergism ratio was calculated by dividing the LD_50_ or LD_95_ of a strain tested with insecticide (Chlorpyrifos-methyl, Pirimiphos-methyl, permethrin or spinosad) alone by the LD_50_ or LD_95_ of the strain tested with insecticide (Chlorpyrifos-methyl, Pirimiphos-methyl, permethrin or spinosad) in combination of either PBO or DEF.

*Significant synergism ratio based on the ratio test, i.e., 95% CI of the ratio did not include 1^32^.

Dose-mortality curves of toxicity of insecticides alone and in combination with synergists are shown in Table 2. Overlapped CI values of LD_50_ and LD_95_, and synergism ratio tests revealed non-significant effect of either synergist on the toxicity of insecticides in the Lab-SO strain. In the case of least susceptible strains to chlorpyrifos-methyl, pirimiphos-methyl and permethrin, both of the synergists significantly increased toxicity of insecticides. Based on non-overlapping 95% CIs and the ratio-test, PBO and DEF significantly reduced LD_50_ values from: 26.93 to 4.59 and 6.38 mg/kg of grains, respectively, for chlorpyrifos-methyl; 29.88 to 3.47 and 4.15 mg/kg of grains, respectively, for pirimiphos-methyl; 41.27 to 26.35 and 23.01 mg/kg of grains, respectively, for permethrin. Similarly, LD_95_ values were also reduced significantly when chlorpyrifos-methyl, pirimiphos-methyl and permethrin were used in combination of either PBO or DEF against the least susceptible field strains of *S. oryzae*. However, none of the synergists could enhance toxicity of spinosad in the least susceptible (FSD-SO) field strain of *S. oryzae*.

**Figure 1:**
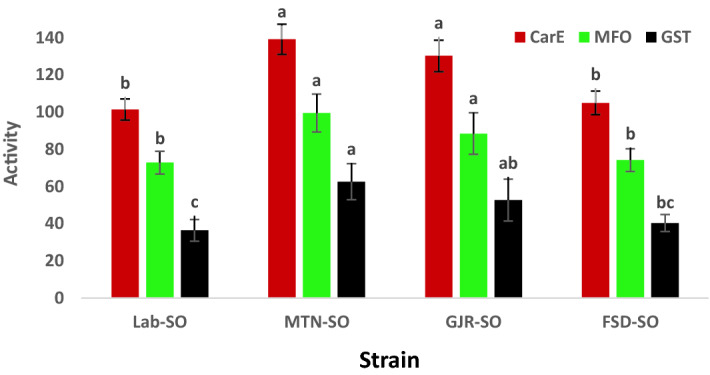
Activities of CarE (nmol/min/mg), MFO (pmol/min/mg) and GST (nmol/min/mg) in the Lab-SO strain and the most resistant strains (MTN-SO, GJR-SO and FSD-SO strains) of *S. oryzae*. Bars of a specific color (mean ± S.E.) with different letters are significantly different based on ANOVA and Tukey’s HSD test.

In addition, significant differences in the activities of CarE (df = 3, 20; F = 39.7; *p* < 0.01), MFO (df = 3, 20; F = 12.7; *p* < 0.01) and GST (df = 3, 20; F = 12.4; *p* < 0.01) were observed in different strains of *S. oryzae*. The MTN-SO and GJR-SO strains exhibited the highest activities of CarE, MFO and GST compared with the Lab-SO strain (Fig. 1).

## Discussion

Management of insect pests using insecticides can only be fruitful if the selection of insecticides is appropriate and insecticides in practice should remain effective against the target pest species. The behavior of insect pests to evolve resistance to commonly used insecticides is one of the major hindrances in the successful pest management programs^8^. For this purpose, variation in toxicity of insecticides should be checked at different intervals in order to make wise decision for successful pest management programs^21^. The current study tried to estimate variation in toxicity of four insecticides, having different modes of action, in laboratory and field strains of *S. oryzae* in Punjab, Pakistan. Chlorpyrifos-methyl and pirimiphos-methyl have the same mode of action in insects i.e., acetylcholinesterase inhibitors. Permethrin is a sodium channel modulator insecticide while the action site of spinosad is nicotinic acetylcholine receptor allosteric modulators^34,35^. The results of the current study showed variable toxicity of all the tested insecticides in different strains of *S. oryzae*. For instance, the Lab-SO strain was the most susceptible strain to all the insecticides tested. The Lab-SO strain showed the highest susceptibility to spinosad followed by permethrin, chlorpyrifos-methyl and pirimiphos-methyl, the latter two were statistically at par. Among the field strains of *S. oryzae*, DGK-SO and FSD-SO were the most susceptible to chlorpyrifos-methyl, while GJR-SO and BWP-SO were the most susceptible strains to pirimiphos-methyl and permethrin, respectively. The BWP-SO strain also showed the highest susceptibility to spinosad in comparison to the rest of the field strains of *S. oryzae*. Moreover, in comparison to the Lab-SO strain at LD_50_ and LD_95_ levels, field strains exhibited: 24.51 to 52.80 and 36.55 to 69.31 resistance ratios, respectively, for chlorpyrifos-methyl; 15.89 to 45.97 and 55.12 to 194.93 resistance ratios, respectively, for pirimiphos-methyl; 39.76 to 108.61 and 61.33 to 130.12 resistance ratios, respectively, for permethrin; 4.23 to 27.50 and 6.28 to 41.00 resistance ratios, respectively, for spinosad. High level of resistance ratios in field strains against chlorpyrifos-methyl, pirimiphos-methyl and permethrin could be linked with long usage history of these insecticides in storage conditions because these insecticides have been in use in Pakistan since 1986, 1982 and 1988, respectively^10^. Recently, laboratory and field strains of *T. granarium* from Punjab, Pakistan, have shown resistance to pirimiphos-methyl and permethrin^4^. The field strains of *T. granarium* exhibited 13.71–24.78 and 13.49–27.94 fold resistance to pirimiphos-methyl and permethrin, respectively, in comparison to a laboratory reference strain at LD_50_ level.

In the present study, resistance to spinosad was relatively low as compared to rest of the insecticides. Previously, very low levels of resistance were reported in field strains of *Tribolium castaneum* (Herbst) (2.24–3.24 fold), *Rhyzopertha dominica* (Fabricius) (3.33–9.00 fold) and *S. oryzae* (1.73–3.45 fold) from Lahore, Jhang, Multan, Sahiwal and Bahawalpur localities of Pakistan. Recently, low levels of resistance to spinosad (2.35–8.77 fold) has also been reported in field strains of *T. granarium* from Punjab, Pakistan^4^. The results of the present study revealed low level of resistance to spinosad in comparison to rest of the insecticides that might be linked with minimal usage of spinosad in storage condition, since spinosad as a grain protectant is not in frequent use in storage facilities in Pakistan. However, spinosad has been extensively used by the farming communities for the management of field-crop pests^4,10^. Hence, there is a probability of selection of resistant individuals due to accidental exposure to insecticide residues under field conditions. In addition, resistance to spinosad could also be due to cross-resistance phenomenon as a result of resistance development against commonly used insecticides^11,15^. Presently, aluminum phosphide, chlorpyrifos-methyl, pirimiphos-methyl, malathion, deltamethrin and permethrin are recommended for the management of stored insect pests in Pakistan^4,10^. Hence, the presence of cross-resistance phenomenon should be figure out in future investigations by selecting spinosad resistance in *S. oryzae* under laboratory conditions.

The present study revealed that field strains of *S. oryzae* collected from different localities exhibited differential response to insecticides. For instance, some strains were more resistant to a particular insecticide while others showed susceptibility or lower resistance to the same insecticide. This behavior probably linked with their history of insecticidal exposures, climate of a particular region, feeding hosts and/or bioassay environment, which made them to respond differently from the strains of other localities^22,36,37^. Studies revealed that susceptibility or resistance status of different strains of the same species could be variable with space and time. For instance, *T. granarium* strains collected from different areas of Punjab, Pakistan, exhibited different responses to spinosad, pirimiphos-methyl and permethrin^4^. Toxicity values (LD_50s_) of *T. granarium* strains ranged from: 17.68–31.97, 20.50–42.47 and 1.34–5.00 mg/kg of grains for pirimiphos-methyl, permethrin and spinosad, respectively. The LC_50_ values of spinosad against different field strains of *T. castaneum*, *R. dominica* and *S. oryzae* collected from Pakistan were ranged from: 0.38–0.45, 0.10–0.27 and 0.19–0.37 mg/kg of grains, respectively^11^. The LD_50_ values of indoxacarb ranged from 0.06–13.99 mg/kg of grains in different field strain of *S. zeamais* (Motschulsky) collected from different localities of Brazil^17^. Recently, we have reported variable toxicity of indoxacarb in different Pakistani field strains of *S. oryzae*, *T. castaneum*, *R. dominica, O. surinamensis* (Linnaeus), and *S. zeamais*^21^. Similarly, variable susceptibilities to cypermethrin, malathion and pirimiphos-methyl were also observed in Egyptian field strains of *T. castaneum* and *S. oryzae*^7^.

Chlorpyrifos-methyl, pirimiphos-methyl, spinosad and permethrin have shown potential to manage different insect pests of stored products. For example, chlorpyrifos-methyl mixed with untreated corn at a concentration of 6 ppm proved effective in controlling populations of *S. zeamais* and *T. castaneum*^38^. Pirimiphos-methyl has recently shown potential to suppress egg hatching and enhance larval mortality of *T. granarium* when applied on concrete surface^39^. In another study, pirimiphos-methyl in the form of capsule suspension exhibited high residual toxicity against *S. granaries*, *T. confusum* and *R. dominica*^40^. Permethrin incorporated netting proved highly effective in the postharvest protection of maize from the attack *S. oryzae*^41^. Similarly, a number of studies have reported efficacy of spinosad in controlling *Cryptolestes ferrugineus* Stephens, *Ephestia kuehniella* (Zeller), *S. oryzae*, *R. dominica, T. castaneum*, *T. confusum*, , *Prostephanus truncatus* (Horn) and *T. granarium*^9,12,36,42^. However, continuous use of insecticides for the management of insect pests usually results in the development of insecticide resistance as have been observed in the current study.

Activation of metabolic detoxifying enzymes has been assumed as one of the major factors responsible for inducing resistance to insecticides^8,24^. The presence of these enzymes in resistant insects can initially be evidenced using combined application of insecticides and synergists in bioassays^15,43^. The synergists used in the present study (PBO and DEF) have the potential to inhibit activities of a number of enzymes mainly responsible for the evolution of resistance to insecticides in insect pests^44^. The results of the present study revealed that both of the synergists significantly suppress resistance to chlorpyrifos-methyl, pirimiphos-methyl and permethrin in field strains (MTN-SO and GJR-SO) of *S. oryzae*, suggesting the probability of metabolic mechanism of resistance. Moreover, both of these strains also showed high activities of CarE, MFO and GST. Previous studies have also reported synergistic effect of PBO or DEF on the toxicity of pirimiphos-methyl in different resistant species^4,45^. However, PBO has also been found to have antagonistic effect on pirimiphos-methyl in *R. dominica*^46^. Similarly, in contrast with the present study, toxicity of pyrethroid insecticide did not increase in synergism experiments with the SzPyrSel strain of the maize weevil^8^. More in vitro investigations can be helpful to further confirm the role of metabolic mechanism of resistance in field strains of *S. oryzae*.

In conclusion, field strains of *S. oryzae* exhibited resistance to all the insecticides. Resistance to spinosad was comparatively at low levels than the rest of the insecticides tested. Synergism studies revealed probable involvement of metabolic mechanism of resistance to insecticides except spinosad. Future research should focus on determining the genetic basis of resistance and the mechanism(s) of resistance in Pakistani strains of *S. oryzae* in order to develop a resistance management framework.

## Data Availability

All data generated or analyzed during this study are included in this published article.
